# Terrestrial Plant- and Algal-Derived Biostimulants as Modulators of ROS and Hormone Networks in Crop Abiotic Stress Resilience

**DOI:** 10.3390/plants15070992

**Published:** 2026-03-24

**Authors:** Pavel Minkov, Tsanko S. Gechev, Aakansha Kanojia

**Affiliations:** 1 Department of Molecular Biology, University of Plovdiv, 4000 Plovdiv, Bulgaria; 2 Department of Molecular Stress Physiology, Center of Plant Systems Biology and Biotechnology, 4023 Plovdiv, Bulgaria

**Keywords:** abiotic stress, antioxidant, hormones, plant-based biostimulant, ROS, seaweed extract

## Abstract

Abiotic stresses severely constrain crop productivity by disrupting cellular redox homeostasis and hormone signaling. Although individual stresses differ in origin, plant responses converge on a conserved regulatory system centered on reactive oxygen species (ROS) and phytohormone crosstalk. Controlled ROS production in chloroplasts, mitochondria and the apoplast functions as a signaling mechanism that interacts dynamically with abscisic acid, auxin, ethylene, jasmonate and cytokinin pathways through shared regulatory nodes, including nicotinamide adenine dinucleotide phosphate (NADPH) oxidases and redox-sensitive transcriptional cascades. Endogenous metabolites, including phenolics, terpenoids, carotenoids, alkaloids, polyamines, glutathione and signaling peptides, are embedded within this network and modulate its amplitude and sensitivity. In parallel, non-microbial biostimulants derived from seaweeds, higher plants, protein hydrolysates and humic substances have been widely reported to enhance crop performance under abiotic stress. However, mechanistic integration between biostimulant research and plant stress signaling remains limited. In this review, we propose that terrestrial plant- and algal-derived biostimulants act not as external substitutes for hormones or antioxidants but as modulators of endogenous ROS–hormone signaling hubs. We first synthesize the current understanding of redox–hormone integration under abiotic stress, then examine endogenous metabolites as intrinsic regulators of this network, followed by an analysis of biostimulants in relation to shared regulatory nodes. By positioning biostimulant action within the established redox–hormone network, we provide a mechanistic framework that links stress biology with agronomic application and supports rational strategies to enhance crop resilience.

## 1. Introduction

Abiotic stresses, including drought, salinity, heat, nutrient limitation, heavy metal contamination and air pollutants, represent major constraints on crop productivity worldwide. The frequency and intensity of several of these stresses are increasing under the current climatic conditions, while limitations on irrigation water and reduced reliance on synthetic inputs are reshaping agricultural management strategies. Improving crop tolerance to abiotic stress is therefore a priority in both high-input and resource-limited production systems [1,2,3].

Plants possess sophisticated mechanisms to perceive and respond to environmental stress. Photosynthesis, respiration, membrane stability, nutrient transport and cellular redox homeostasis are consistently affected. Disruption of these processes leads to enhanced production of reactive oxygen species (ROS) in chloroplasts, mitochondria, peroxisomes and the apoplast [4]. At controlled concentrations, ROS function as second messengers that regulate gene expression, protein activity and systemic signaling [5,6]. When accumulation exceeds buffering capacity, ROS cause oxidative damage to lipids, proteins and nucleic acids [5,6]. In parallel, abiotic stresses induce rapid and dynamic changes in phytohormone biosynthesis, transport and sensitivity [5]. Abscisic acid (ABA), auxins, cytokinins, ethylene, jasmonates, salicylic acid (SA) and brassinosteroids collectively regulate stomatal conductance, root system architecture, growth restraint, osmotic adjustment and defense-related transcription [5,7,8]. Increasing evidence indicates that ROS and hormone pathways do not operate independently. Instead, they form an interconnected regulatory network in which ROS influence hormone biosynthesis and signaling while hormones modulate ROS production and antioxidant capacity [5,9,10]. Shared regulatory nodes include NADPH oxidases (respiratory burst oxidase homologs, RBOHs), mitogen-activated protein kinase (MAPK) cascades, redox-sensitive transcription factors and thiol-dependent regulatory proteins [5,11]. This integrated ROS–hormone architecture underpins cross-stress tolerance and priming responses [5,12].

Endogenous plant metabolites and signaling peptides are closely linked to this regulatory network. Phenolic compounds, terpenoids, carotenoids, alkaloids, polyamines, glutathione and small secreted peptides contribute not only to ROS scavenging but also to modulation of ROS production, hormone sensitivity and redox-dependent transcription [13,14,15,16]. These metabolites converge on common regulatory nodes, adjusting the amplitude and spatial distribution of stress signals rather than functioning solely as terminal antioxidants. Thus, abiotic stress adaptation can be understood as quantitative regulation of a pre-existing redox–hormone network [13,14]. In parallel with advances in stress signaling research, agricultural use of plant biostimulants has expanded rapidly. Under Regulation (EU) 2019/1009, plant biostimulants are defined as products that stimulate plant nutrition processes independently of nutrient content with the aim of improving nutrient use efficiency, stress tolerance, crop quality or nutrient availability in the rhizosphere [17,18]. Non-microbial biostimulants include seaweed extracts, higher plant extracts, protein hydrolysates (PHs) and humic and fulvic substances. Numerous studies report improvements in growth, yield and stress indicators following the application of these products [19,20,21,22]. However, despite extensive agronomic documentation, mechanistic integration between biostimulant research and plant stress signaling remains incomplete. Many studies describe enhanced antioxidant enzyme activity or altered hormone levels, but few place these responses within the established ROS–hormone regulatory framework that governs endogenous stress adaptation. As a result, biostimulant effects are often interpreted descriptively rather than mechanistically.

We address this gap by proposing that terrestrial plant-derived and algal-derived biostimulants act as external modulators of the same ROS–hormone signaling hubs that regulate intrinsic stress responses. In this review, “terrestrial plant-derived” refers to extracts obtained from vascular land plants, whereas “algal-derived” encompasses products obtained from marine macroalgae (seaweeds) and microalgae. First, we summarize the common physiological and molecular features of major abiotic stresses, emphasizing redox imbalance and hormone crosstalk. Second, we examine endogenous plant metabolites and signaling peptides as integral regulators of ROS–hormone networks. Third, we analyze non-microbial biostimulants of terrestrial plant and algal origin and interpret their reported physiological and molecular effects within this established regulatory architecture. By integrating stress biology with biostimulant research, we aim to shift the focus from descriptive “antioxidant enhancement” towards a network-based interpretation in which both endogenous metabolites and exogenous biostimulants tune shared redox–hormone regulatory nodes. This perspective provides a mechanistic framework for evaluating biostimulant efficacy and designing targeted strategies to enhance abiotic stress tolerance in crops.

## 2. Abiotic Stress, ROS, Hormones and Plant Stress Adaptation—An Integrative Framework

Crop plants experience diverse abiotic stresses, such as heat, cold, drought, salinity, waterlogging, heavy metals, and air pollutants. Although these stresses differ in their primary triggers, they converge on a limited set of core physiological processes. A common outcome is disruption of photosynthetic efficiency, membrane stability, water retention, nutrient homeostasis, cellular redox balance and hormone regulation [23,24,25]. Disruption of these processes frequently results in enhanced production of reactive oxygen species (ROS) and hormonal reprogramming [26,27,28,29].

A key outcome of abiotic stress is the activation of interconnected ROS–hormone signaling hubs. These hubs integrate environmental signals and coordinate downstream responses, including antioxidant activation, metabolic reprogramming, stomatal regulation and growth adjustment. In the following subsections, we focus on the main regulatory nodes in these hubs, plasma membrane NADPH oxidases (respiratory burst oxidase homologues, RBOHs), mitogen-activated protein kinase (MAPK) cascades, calcium-signaling modules and redox-sensitive transcription factors, which together couple ROS production with hormone-dependent regulatory pathways.

### 2.1. ROS and Hormone Signaling Under Abiotic Stress

ROS are continuously produced in chloroplasts, mitochondria and peroxisomes during aerobic metabolism and increase under stress [30]. The main ROS radicals include superoxide (O_2_•^−^), hydrogen peroxide (H_2_O_2_), singlet oxygen (^1^O_2_) and hydroxyl radicals (•OH) [5]. In addition to organelles, the apoplast generates ROS via plasma membrane NADPH oxidases known as RBOHs [31]. In chloroplasts, restricted CO_2_ fixation promotes electron transfer to oxygen, generating O_2_•^−^ and ^1^O_2_. In mitochondria, electron leakage from the respiratory chain produces O_2_•^−^ when ATP demand or oxygen supply is disturbed. Peroxisomes generate H_2_O_2_ during photorespiration and β-oxidation [5,30]. In the apoplast, RBOHs produce O_2_•^−^, which is rapidly converted to H_2_O_2_ and can diffuse into cells or modify cell-wall components [11]. At high concentrations ROS cause oxidative damage; however, controlled ROS production functions as a signaling mechanism [5]. H_2_O_2_ can diffuse across membranes and modify cysteine residues in target proteins, thereby regulating kinases, phosphatases and transcription factors. At high and uncontrolled levels, ROS oxidize lipids, proteins and nucleic acids, leading to membrane leakage, enzyme inactivation and DNA damage [32,33].

To prevent ROS overproduction, plants maintain enzymatic antioxidants, such as superoxide dismutase (SOD), catalase (CAT), ascorbate peroxidase (APX), peroxidase (POD) and glutathione reductase, and non-enzymatic antioxidants, including ascorbate, glutathione, tocopherols, carotenoids and phenolic compounds [34]. Together, these systems limit ROS overproduction and maintain redox homeostasis. However, under many abiotic stresses, ROS accumulate and function not only as damaging agents but also as signaling molecules that regulate gene expression, metabolism, and plant growth [5,30]. Hormones also regulate antioxidant capacity, where ABA, SA and jasmonates modulate the expression of genes encoding SOD, APX and glutathione-related enzymes. In turn, redox status influences hormone biosynthesis and signaling sensitivity [30]. A central example of ROS–hormone integration occurs in guard cells. ABA activates SnRK2 kinases, which stimulate RBOH activity and promote H_2_O_2_ accumulation. This ROS signal activates Ca^2+^ channels and downstream responses, leading to stomatal closure [35,36]. Thus, ROS function downstream and upstream of ABA signaling. Redox-sensitive transcriptional regulators integrate hormonal and ROS signals, where non-expressor of PR Gene 1 (*NPR1*), a key regulator of SA signaling, is activated through redox-dependent thiol modification [37,38]. MAPK cascades and calcium-dependent protein kinases (CDPKs) are also modulated by ROS and participate in ABA, jasmonate and ethylene pathways [39]. ROS signaling and systemic signaling further illustrate integration across tissues, where local stress can induce rapid systemic ROS production mediated by RBOHD, contributing to systemic acquired acclimation [39]. Moreover, phytoglobins (PGBs) act as important redox buffers and modulators of ROS–NO signaling by scavenging nitric oxide (NO), which often leads to a reduced ROS level and decreased expression of RBOH genes, effectively preventing the signal from reaching toxic levels [40]. Different cellular compartments generate distinct ROS “signatures” in terms of species, concentration and timing, and these signatures are integrated with hormone gradients and sensitivities. Drought, salinity and temperature stresses all increase ROS, but in different organelles and with different kinetics, and they induce specific combinations of hormonal changes [41,42]. The integration of these ROS and hormone patterns shapes downstream responses, including stress gene expression, growth adjustments and stress adaptation [5,30].

The concept of ROS and hormone homeostasis therefore includes not only the balance between their production and removal but also the regulation of their signaling outputs. When this balance is maintained, ROS and hormones act as coordinated signals that promote acclimation, for example by inducing antioxidants, osmolyte synthesis, cell-wall reinforcement and adjustments in root and shoot development [6,43]. When the balance is lost, excessive ROS and imbalanced hormones contribute to oxidative damage, growth inhibition and premature senescence [6,43]. Understanding this dynamic integration of ROS and hormone signaling is essential for interpreting how plant-derived biostimulants may modulate stress responses as many biostimulant components may influence the ROS–hormone signaling hubs.

### 2.2. Cross-Stress Interactions and Priming Build Endogenous Stress Adaptive Memory

In natural and agricultural environments, plants often experience combinations of stresses, such as drought with heat or salinity with nutrient limitation. Responses to one stress can therefore influence responses to others, a phenomenon described as cross-stress interaction or cross-tolerance [12,44,45,46]. These interactions may be beneficial, where exposure to one stress increases tolerance to another, or detrimental, where prior stress increases sensitivity. Cross-tolerance is frequently mediated by shared signaling components, including ROS and hormones [4,12,44]. Transient ROS production under one stress can activate antioxidant systems and defense genes that also provide protection against other stresses [44]. Similarly, ABA, jasmonates, SA, ethylene and brassinosteroids regulate sets of genes and metabolic pathways that are relevant for multiple stresses. For example, mild drought can induce changes in stomatal movement, root architecture and antioxidant capacity, together with increased ABA, altered cytokinin and ethylene levels, which, collectively, can improve performance during later heat or salinity episodes [5,12,44].

Priming is a related phenomenon in which a prior stimulus, such as mild abiotic stress, a chemical signal or a biological agent, places the plant in a physiological state that allows faster or stronger activation of adaptive responses upon subsequent stress [47,48,49]. Primed plants often show earlier and higher induction of antioxidant enzymes, more rapid accumulation of osmoprotectants and more robust activation of stress-responsive transcription factors than non-primed plants [47,48,49]. Hormones play an important role in priming. Low doses of ABA, SA, jasmonates, ethylene or brassinosteroids, as well as combinations of these, can induce a primed state without causing major damage or growth inhibition [47,50]. Molecular studies indicate that priming involves chromatin modifications, changes in redox status and the accumulation of “dormant” defense proteins and signaling components that are activated more quickly when stress occurs [47,49]. Endogenous secondary metabolites and peptides contribute to these processes. Phenolic compounds, terpenoids, alkaloids and sulfur-containing metabolites can interact with ROS and hormone pathways, strengthen cell walls and modulate signaling cascades [48]. Small peptides and peptide hormones participate in stress perception, long-distance communication and coordination of growth with stress responses [13,15,16].

**Figure 1:**
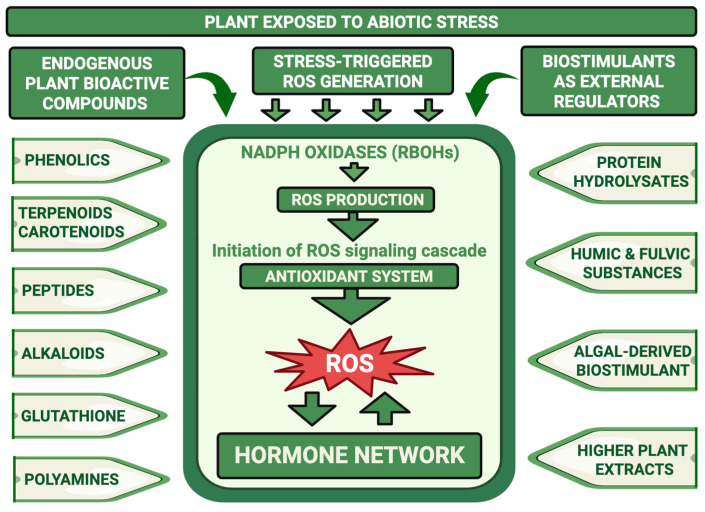
Conceptual framework integrating ROS–hormone signaling hubs as common regulatory targets of endogenous plant bioactive compounds and biostimulants under abiotic stress. Abiotic stresses disrupt cellular redox homeostasis, leading to activation of NADPH oxidases (RBOHs), organellar ROS production, antioxidant systems, and hormone signaling pathways. Endogenous plant bioactive compounds modulate these regulatory nodes, contributing to stress acclimation and priming. Terrestrial plant- and algae-derived biostimulants, acting as external regulators, operate within this pre-existing network by influencing ROS generation and scavenging, as well as hormone biosynthesis and signaling components. The figure was created using BioRender.com. Rai, A. (2026).

Together, these ROS–hormone signaling hubs, cross-stress interactions and priming mechanisms define an endogenous adaptive network that allows plants to integrate information about past and present stresses [51]. This network is directly relevant for understanding how plant-derived biostimulants act because many biostimulant products contain molecules that resemble endogenous secondary metabolites, hormones or signaling peptides, or they influence ROS and hormone balances [51]. To integrate this view, Figure 1 presents our conceptual framework: abiotic stress triggers ROS production in organelles and the apoplast, which is processed by antioxidant systems and integrated with hormone signaling nodes. Endogenous bioactive compounds and externally applied biostimulants both target these shared ROS–hormone hubs, altering the amplitude, timing and spatial distribution of stress signals rather than creating new signaling pathways. In Section 3, we therefore focus on endogenous plant bioactive compounds and their roles in regulating ROS–hormone networks to provide a mechanistic baseline for stress adaptation. Section 4 then builds on this framework by examining how terrestrial-derived and algal-derived biostimulants modulate the same signaling modules and stress-adaptive networks to enhance tolerance to abiotic stresses.

## 3. Endogenous Plant Bioactive Compounds as Regulators of ROS and Hormone Networks

As summarized in the conceptual framework of Figure 1, plants accumulate diverse endogenous metabolites and peptides that contribute to stress perception and adaptive reprogramming under abiotic stress [2,52,53]. Importantly, many of these compounds do not function solely as ROS scavengers. Instead, they modulate ROS production, redox buffering capacity, hormone signaling, and cell-wall signaling pathways [54,55]. Emerging evidence indicates that these endogenous bioactive molecules converge on shared regulatory nodes, including NADPH oxidases (RBOHs), redox-sensitive transcription factors and hormone signaling cascades. In the following sections, we examine major classes of plant metabolites and signaling molecules that integrate ROS and hormone networks: phenolic compounds, terpenoids and carotenoids, alkaloids, small signaling peptides, glutathione and sulfur metabolism, and polyamines.

### 3.1. Phenolic Compounds as Regulators of ROS and Hormone Signaling

Phenolic compounds, including phenolic acids, flavonoids and tannins, are among the most abundant secondary metabolites in plants and are primarily synthesized via the phenylpropanoid pathway [56]. Their accumulation is strongly induced under drought, salinity, temperature extremes and heavy metal stress, where they contribute to both antioxidant protection and stress signaling [57,58]. Enhanced flux through the phenylpropanoid pathway under stress is associated with increased activity of phenylalanine ammonia-lyase (PAL) and coordinated transcriptional activation of genes encoding cinnamate 4-hydroxylase (C4H), 4-coumarate:CoA ligase (4CL), chalcone synthase (CHS), chalcone isomerase (CHI), flavanone 3-hydroxylase (F3H), dihydroflavonol 4-reductase (DFR), flavonol synthase (FLS) and related enzymes [58,59,60,61]. This transcriptional reprogramming increases the diversity and accumulation of flavonoids and related phenolics during stress adaptation.

**Figure 2:**
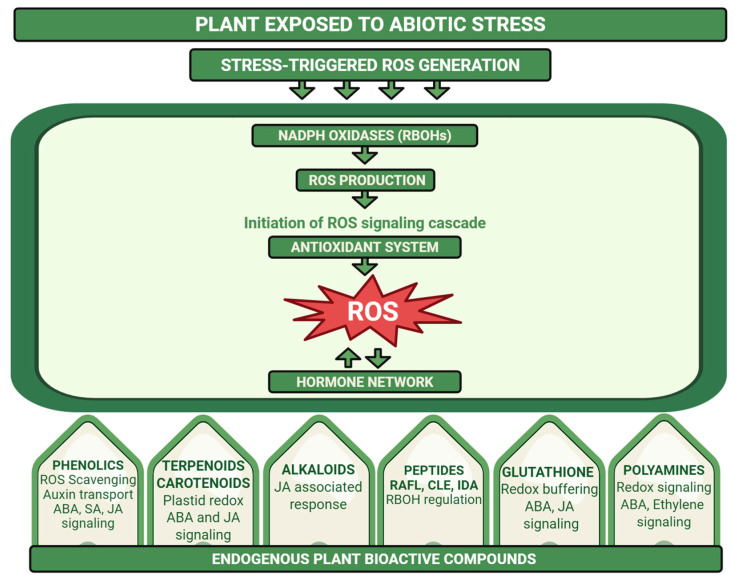
Endogenous plant bioactive compounds as regulators of ROS–hormone signaling networks under abiotic stress. Abiotic stress disrupts cellular redox homeostasis, leading to the activation of reactive oxygen species (ROS) production through NADPH oxidases (RBOHs), alongside activation of antioxidant systems and phytohormone signaling pathways. Endogenous plant bioactive compounds function as intrinsic regulators within this network by modulating ROS generation and scavenging, as well as hormone biosynthesis and signaling components.

**Figure 3:**
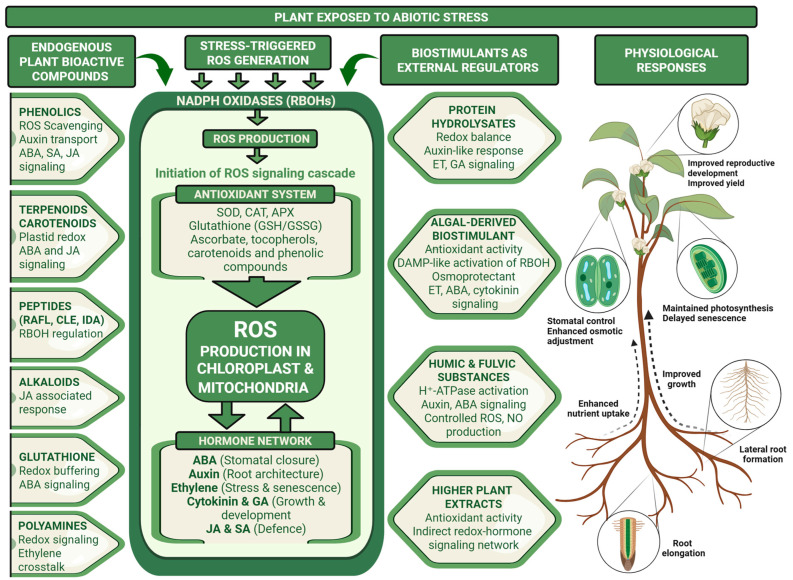
Integrated model of ROS–hormone signaling hubs as common regulatory targets of endogenous plant bioactive compounds and biostimulants under abiotic stress. Abiotic stresses disrupt cellular redox homeostasis, leading to activation of NADPH oxidases (RBOHs), organellar ROS production, antioxidant systems, and hormone signaling pathways (ABA, auxin, ethylene, cytokinin, GA, JA, and SA). Endogenous secondary metabolites and peptide signals modulate these same regulatory nodes, contributing to stress acclimation and priming. Terrestrial plant- and algae-derived biostimulants, acting as external regulators, operate within this pre-existing network by influencing ROS generation and scavenging, as well as hormone biosynthesis and signaling components. Downstream physiological outputs include stomatal regulation, adjustment of root system architecture, delayed senescence, maintenance of photosynthetic performance, improved growth, reproductive development and yield stability. The model emphasizes convergence on shared regulatory hubs rather than activation of independent signaling pathways. ABA, abscisic acid; JA, jasmonic acid; GA, gibberellin; SA, salicylic acid. The figure was created using BioRender.com. Rai, A. (2026).

Flavonoids act as spatially localized ROS scavengers in chloroplasts and epidermal tissues, limiting oxidative damage under high light and drought [57,62]. Their accumulation correlates with reduced lipid peroxidation and improved membrane stability under salinity and metal stress, often in association with enhanced activity of enzymatic antioxidants [58,62]. Beyond ROS detoxification, phenolic metabolism is tightly integrated with hormone signaling. Flavonoids regulate auxin distribution by modulating PIN-FORMED (PIN) auxin efflux carriers, thereby influencing root architecture under stress [63]. ABA signaling induces PAL and CHS expression during drought, linking hormone-dependent transcriptional control with enhanced redox buffering [64]. In addition, jasmonate and SA pathways intersect with phenylpropanoid metabolism to coordinate oxidative and defense responses [65,66]. Phenolics also contribute structurally to stress adaptation through lignin deposition and phenolic cross-linking in the cell wall, processes that restrict pathogen entry and reduce cellular damage [67,68]. Together, these findings demonstrate that phenolic compounds operate at the interface of ROS control, hormone signaling and structural reinforcement rather than functioning solely as passive antioxidants. Within our conceptual framework (Figure 1), phenolics can therefore be viewed as endogenous modulators of the ROS–hormone hub, tuning RBOH-derived ROS production, antioxidant buffering capacity and hormone signaling outputs that are also targeted by plant-derived biostimulants (Figure 2 and Figure 3).

### 3.2. Terpenoids and Carotenoids Connect ROS, Hormones and Volatile Signaling

Terpenoids comprise a diverse group of metabolites, including volatile isoprene and monoterpenes as well as carotenoids. A growing body of evidence shows that several terpenoids contribute directly to redox balance under abiotic stress. Isoprene, for example, has been shown to enhance tolerance to heat and oxidative stress by stabilizing membranes and limiting ROS accumulation [69]. Transgenic tobacco plants engineered to emit isoprene exhibit reduced ozone-induced ROS accumulation and lipid peroxidation compared with non-emitting lines [70]. In addition to direct antioxidant effects, volatile terpenoids function as signaling molecules. Jasmonic acid and methyl jasmonate induce terpene synthase genes and volatile emission [71]. Conversely, stress-induced volatiles can prime defense responses in neighboring plants, indicating feedback regulation between ROS production, jasmonate signaling and airborne communication [72].

Carotenoids are central to chloroplast redox control. The xanthophyll cycle regulates non-photochemical quenching (NPQ) and limits ^1^O_2_ formation in photosystem II [73]. *Arabidopsis (Arabidopsis thaliana)* mutant NPQ deficient in zeaxanthin formation shows impaired NPQ and increased oxidative damage under high light [74], confirming the role of carotenoids in ROS management. Carotenoids also serve as precursors of key hormones. The 9-cis-epoxycarotenoid dioxygenase (NCED)-mediated cleavage of 9-cis-epoxycarotenoids represents a rate-limiting step in ABA biosynthesis [75], and drought-induced NCED expression leads to ABA accumulation and activation of stress responses [76]. Carotenoid-derived strigolactones further regulate shoot branching and root development under nutrient and water limitation [77,78]. Collectively, terpenoid metabolism links plastid redox state with ABA biosynthesis, jasmonate-dependent signaling and volatile-mediated communication, reinforcing its central position within the ROS–hormone network. In the ROS–hormone hub (Figure 1), terpenoids and carotenoids thus function as plastid-based regulators that couple ROS homeostasis to ABA/JA signaling and volatile-mediated communication, thereby modulating both the intensity of ROS signals and the downstream hormonal outputs that are also targeted by biostimulants (Figure 2 and Figure 3).

### 3.3. Alkaloids as Modulators of Jasmonate-Associated Redox Responses

Alkaloids are nitrogen-containing secondary metabolites widely known for their roles in defense against herbivores and pathogens [79]. Increasing evidence indicates that alkaloid biosynthesis is integrated into stress-responsive hormone networks, particularly jasmonate signaling. In tobacco, jasmonic acid induces key nicotine biosynthetic genes [80]. Moreover, in *Catharanthus roseus*, jasmonate treatment induces terpenoid indole alkaloid biosynthesis genes, including strictosidine synthase, demonstrating hormonal regulation of alkaloid production [81]. Alkaloid accumulation under drought and salinity has been associated with reduced lipid peroxidation and enhanced antioxidant enzyme activity. In halophytic and medicinal species, increased alkaloid levels have been associated with lower lipid peroxidation and enhanced activity of antioxidant enzymes [82]. Although mechanistic resolution remains limited compared with other metabolite classes, these observations suggest that alkaloids contribute to redox stabilization during stress. Because alkaloid biosynthesis depends on nitrogen allocation, shifts in nitrogen metabolism may indirectly influence other redox-associated pathways, including polyamine metabolism. Thus, alkaloids likely participate in jasmonate-associated redox regulation rather than functioning solely as endpoint defense compounds. Within our ROS–hormone hub framework (Figure 1), alkaloids can therefore be viewed as JA-responsive metabolites that feed back on redox status by modulating antioxidant capacity and lipid peroxidation, thereby adjusting the intensity and outcomes of jasmonate-linked ROS signaling (Figure 2 and Figure 3).

### 3.4. Small Signaling Peptides Integrate ROS and Hormones

Small secreted peptides form an additional regulatory layer linking cell-wall integrity, ROS production and hormone signaling [83]. Rapid Alkalinization Factor (RALF) peptides are among the best-characterized stress-related peptides. They are perceived by the receptor-like kinase FERONIA (FER), which regulates NADPH oxidase activity and ROS production [84,85]. In *Arabidopsis*, *fer* mutants display increased salt sensitivity, altered ROS accumulation and impaired ion homeostasis, demonstrating the functional integration of peptide perception with redox and stress signaling [81]. CLE (CLAVATA3/EMBRYO SURROUNDING REGION-related) peptides are best known for regulating meristem activity, and they also contribute to stress-dependent root regulation. Under phosphate deficiency, CLE14 expression is strongly induced in *Arabidopsis* root tips. Gutiérrez-Alanís et al. showed that CLE14 promotes premature root meristem differentiation through the CLAVATA2 (CLV2) receptor complex and requires ROS accumulation in the proximal meristem [86]. Phosphate starvation alters ROS distribution and auxin responses in the root meristem [87], indicating that CLE-mediated signaling integrates nutrient sensing, ROS accumulation and hormone regulation. IDA (INFLORESCENCE DEFICIENT IN ABSCISSION) and IDA-like peptides further modulate ROS bursts during developmental and stress processes [88]. Because ROS waves contribute to systemic stress signaling [31], peptide-mediated control of NADPH oxidase activity may influence whole-plant acclimation. Collectively, small peptides operate as receptor-mediated regulators of redox and hormone signaling. In our conceptual hub (Figure 1), RALF–FER, CLE–CLV2 and IDA pathways sit directly upstream of RBOH-derived ROS, Ca^2+^ fluxes and hormone responses, positioning stress-responsive peptides as extracellular inputs that tune ROS–hormone signals (Figure 2 and Figure 3).

### 3.5. Glutathione and Sulfur Metabolism in Redox–Hormone Integration

Glutathione (GSH) is a central regulator of plant redox homeostasis and stress signaling, linking sulfur assimilation to ROS control [89,90]. The GSH/GSSG ratio modulates H_2_O_2_ turnover and thiol-dependent regulation of proteins involved in stress signaling [89]. For instance, in *Arabidopsis*, the glutathione-deficient mutant *rax1-1* shows altered expression of oxidative stress-responsive genes, indicating that glutathione status influences redox-dependent transcriptional regulation [91]. Munemasa et al. further demonstrated that glutathione-deficient mutant *cad2-1* in *Arabidopsis* glutathione affects ABA-induced stomatal closure and ROS accumulation, demonstrating functional interaction between redox buffering and ABA signaling [92]. Sulfur assimilation also responds to oxidative and hormonal cues, where expression of genes encoding enzymes such as γ-glutamylcysteine synthetase increases under abiotic stress, supporting enhanced GSH synthesis [93]. Sulfur metabolism is further regulated by jasmonates and oxidative signals, highlighting the integration of redox buffering, hormonal control, and stress adaptation [93,94]. Through modulation of redox-sensitive proteins and hormone responses, glutathione functions not only as an antioxidant but also as a signaling integrator within the ROS–hormone network. In summary, glutathione and sulfur metabolism shape the redox background of the ROS–hormone hub, so changes in GSH status directly affect how RBOH-derived ROS and ABA/JA signaling are interpreted and how stress responses can be reprogrammed by endogenous metabolites or biostimulants (Figure 2 and Figure 3).

### 3.6. Polyamines in ROS and Hormone Crosstalk

Polyamines, including putrescine, spermidine and spermine, accumulate under abiotic stress and are closely associated with ROS metabolism. Their oxidation generates H_2_O_2_, contributing to local and systemic redox signaling [95]. Polyamines enhance antioxidant enzyme activity, stabilize membranes, and regulate stress-responsive gene expression [96,97]. Studies have shown that exogenous application of spermidine or spermine enhances activities of SOD, CAT and POD under salinity and drought conditions [96]. Polyamine metabolism intersects directly with hormone pathways. For instance, S-adenosylmethionine is a shared precursor for polyamine and ethylene biosynthesis, and metabolic flux influences ethylene production [98]. Polyamines also modulate ABA-induced stomatal closure and ion channel activity in guard cells, further linking them to redox- and hormone-regulated processes. Additionally, polyamines affect NADPH oxidase activity and ROS production, placing them at a convergence point between redox signaling and hormonal regulation [99,100]. Through coordinated effects on H_2_O_2_ production, antioxidant capacity, ethylene biosynthesis and ABA responsiveness, polyamines act as dynamic regulators of the ROS–hormone network during abiotic stress adaptation. In the framework of Figure 1, polyamines interface with the hub at multiple points by feeding H_2_O_2_ into RBOH- and PAO-dependent ROS circuits, modulating ion channels and guard-cell signaling, and sharing precursors with ethylene, thereby providing endogenous control points that external biostimulants may target to regulate ROS–hormone dynamics (Figure 2 and Figure 3).

## 4. Terrestrial Plant- and Algal-Derived Biostimulants as External Regulators of ROS–Hormone Networks

Building on the conceptual framework in Figure 1, plants possess an intrinsic network of metabolites and signaling molecules that control ROS production, redox buffering and hormone responses under abiotic stress, as outlined in Section 2 and Section 3. Central regulatory nodes include NADPH oxidases, antioxidant enzymes and ABA-, auxin-, jasmonate- and ethylene-dependent signaling cascades. A key question, therefore, is whether externally applied biostimulants activate independent pathways or operate through the same endogenous regulatory framework.

The term “plant biostimulant” has been formally defined as a substance or microorganism that stimulates plant nutrition processes independently of nutrient content, with the aim of improving nutrient use efficiency, stress tolerance or crop quality [19,101]. Among non-microbial biostimulants, seaweed extracts, humic substances, PH and higher plant extracts are the most studied categories. Although our review focuses on these non-microbial products, several studies indicate that seaweed extracts, PH and humic-based formulations can also act indirectly by reshaping the rhizosphere microbiome. Applications of *Ascophyllum nodosum* extracts, for example, have been shown to modify bacterial and fungal communities around roots and to increase root and shoot biomass [102], while PH and humic substances provide carbon substrates that favor plant-growth-promoting taxa capable of producing indole-3-acetic acid (IAA) or 1-aminocyclopropane-1-carboxylate (ACC) deaminase, thereby influencing nutrient availability and hormone homeostasis [103,104,105]. These microbiome-mediated effects represent an additional indirect route through which non-microbial biostimulants can feed back into root-associated ROS–hormone networks, even though microbial biostimulants per se are outside the scope of this review.

In our model (Figure 1 and Figure 3), these biostimulants are viewed as external inputs that target existing ROS–hormone hubs, for example by modulating RBOH activity, antioxidant capacity or the hormone-responsive transcriptional network, rather than as sources of novel signaling pathways. Accumulating evidence indicates that terrestrial plants and algal-derived biostimulants do not introduce novel signaling systems. Instead, they influence ROS production, antioxidant capacity and hormone-responsive gene expression within existing plant signaling networks. For example, extracts from the brown alga *Ascophyllum nodosum* enhance drought and salinity tolerance by increasing antioxidant enzyme activity and modulating ABA-responsive gene expression [106]. Transcriptomic analyses show altered expression of stress-related transcription factors and ROS-scavenging genes following seaweed extract application. Humic substances have been shown to stimulate lateral root formation through auxin-like activity and modulation of H^+^-ATPase activity [107,108]. In addition, humic fractions can induce transient ROS production and activate plasma membrane redox systems, suggesting interaction with RBOH-mediated signaling [109]. Plant-derived extracts and PH contain peptides and amino acids that regulate nitrogen metabolism and stress-related pathways [110]. Similarly, extracts derived from higher terrestrial plants, such as *Moringa oleifera* and *Medicago sativa* (alfalfa), enhance stress tolerance by enhancing antioxidant activity and modulating redox–hormone-associated signaling pathways [111,112]. A comparative overview of these non-microbial biostimulant categories, their principal bioactive constituents, regulatory targets within ROS–hormone networks, and associated physiological effects under abiotic stress is presented in Table 1 and Figure 3, while Table 2 summarizes the species of terrestrial plants and algae used in biostimulant production and their documented effects on crops.

Taken together, the current evidence supports the view that plant- and algae-derived biostimulants act as external inputs into pre-existing ROS–hormone signaling networks. Their effects can be interpreted as quantitative adjustments in ROS amplitude, antioxidant buffering capacity and hormone sensitivity rather than activation of independent signaling circuits. The following subsections analyze mechanistic data for major biostimulant classes, with an emphasis on their interaction with NADPH oxidases, antioxidant systems and hormone-dependent pathways.

### 4.1. Terrestrial Plant-Derived Protein Hydrolysates and Redox–Hormone Regulation

Protein hydrolysates (PHs) are complex mixtures of short peptides (typically 2–20 amino acids), free amino acids and low-molecular-weight organic compounds generated through enzymatic hydrolysis of plant biomass [110]. Although initially described as growth-promoting inputs, increasing physiological, transcriptomic and metabolomic evidence indicates that PHs act as signaling modulators that integrate into endogenous ROS and hormone regulatory networks rather than functioning solely as nutrient supplements [110].

Across multiple crops and stress contexts, PH application consistently alters redox balance. Under salinity, vegetal PH treatment in lettuce and tomato enhanced the activity of key antioxidant enzymes, including SOD, CAT, APX and POD, while reducing lipid peroxidation [116,117]. Similar effects were reported under heat stress in maize, where PH-treated plants showed reduced malondialdehyde (MDA) accumulation and increased proline content, indicating improved cellular redox stability [118]. Importantly, these responses reflect modulation of ROS homeostasis rather than simple ROS scavenging. In stress signaling, ROS generated by NADPH oxidases (RBOHs) function as second messengers that activate MAP kinase cascades and redox-sensitive transcription factors. Enhancement of antioxidant capacity by PHs likely reshapes the amplitude and duration of these ROS signals, preventing oxidative damage while preserving signaling competence. Thus, PH treatment appears to shift the redox set-point of stressed tissues toward controlled ROS accumulation compatible with signal transduction rather than oxidative injury.

Evidence from metabolomic analyses further supports systemic reprogramming. In tomato subjected to repeated drought cycles, application of the plant-derived PH ‘Trainer^®^’ altered the dipeptide pools, fatty acid composition and phenolic metabolism, and improved biomass recovery after successive stress events [119]. These metabolic shifts resemble endogenous acclimation patterns, where accumulation of compatible solutes, membrane remodeling and activation of secondary metabolism stabilize cellular structures under stress. Similarly, transcriptomic studies in maize revealed that PH treatment modulated genes associated with redox homeostasis, stress-responsive pathways, flavonoid and terpenoid biosynthesis, and phytohormone signaling [120]. In tomato, PH application upregulated genes linked to photosynthesis, detoxification processes and nitrogen metabolism [121]. Collectively, these findings indicate that PHs induce coordinated metabolic reconfiguration involving ROS detoxification systems, secondary metabolite biosynthesis and hormone-related transcriptional programs rather than isolated enhancement of single enzymes.

Several studies demonstrate hormone-like activities of PHs, particularly related to auxin and gibberellin pathways. Application of PH ‘Trainer^®^’ to gibberellin-deficient pea increased shoot length, suggesting gibberellic acid (GA)-like activity [116]. In maize, collagen-derived PH markedly stimulated lateral root growth and absorptive root area, with low-molecular-weight peptide fractions being particularly active [122]. Auxin-regulated root development is tightly linked to localized ROS gradients in the root meristem, where controlled ROS production influences cell division and differentiation. Therefore, PH-induced changes in root architecture likely involve coordinated modulation of ROS–auxin crosstalk, potentially through regulation of redox-dependent auxin transport and signaling components. In this context, PHs may influence root system architecture by adjusting both hormonal cues and redox microenvironments within meristematic tissues. Moreover, recent multi-omics evidence extends PH action to ethylene-associated signaling. Metabolomic profiling of PH-treated tomato plants revealed increased accumulation of ACC, the immediate precursor of ethylene biosynthesis and polyamines [119]. Under nitrogen limitation, integrated transcriptomic analysis showed upregulation of genes encoding ethylene receptors (ETR/ERS) and ACC oxidase in tomato treated with a Malvaceae-derived PH [123], indicating modulation of ethylene signaling components. Ethylene biosynthesis and polyamine production share the common precursor S-adenosyl-L-methionine (SAM), creating a metabolic node that integrates stress-induced shifts in nitrogen status, redox balance and hormone signaling. Both ethylene and polyamines interact with ROS signaling. Ethylene can regulate NADPH oxidase activity and stress-responsive transcription factors, while polyamines influence ROS production and scavenging dynamics. PH-induced changes in ACC levels and ethylene-related gene expression therefore suggest modulation of this SAM-dependent regulatory hub. Rather than directly increasing ethylene output, PHs appear to fine-tune the balance between ethylene biosynthesis, receptor signaling and associated redox responses.

Taken together, physiological, metabolomic and transcriptomic evidence supports a model in which plant-derived PHs act as external peptide-rich signals that integrate into endogenous ROS–hormone networks. They modify antioxidant capacity, reshape secondary metabolism, and influence auxin-, gibberellin- and ethylene-associated pathways through shared redox-sensitive regulatory nodes. Thus, PHs should not be viewed merely as growth enhancers or nutrient supplements. Instead, they function as modulators of the existing redox–hormone signaling architecture, influencing how stress signals are generated, transmitted and resolved under adverse environmental conditions (Figure 3).

### 4.2. Algae-Derived Biostimulants and Redox–Hormone Regulation

Algae-derived seaweed extracts are obtained primarily from brown (Phaeophyceae), red (Rhodophyceae), and green (Chlorophyceae) macroalgae, each group containing distinct bioactive compounds that exert specific physiological and molecular effects on plants [124]. These extracts typically contain complex polysaccharides, such as alginates, laminarins and fucoidans, along with betaines, phenolics, trace minerals and hormone-like compounds [124]. Rather than acting as simple nutrient supplements, these components function synergistically to influence root development, nutrient uptake, osmotic adjustment and antioxidant systems [125]. Seaweed-derived polysaccharides, particularly alginates and laminarins, can function as signaling molecules that stimulate meristem activity and root branching, thereby improving nutrient and water acquisition [126]. Amino acid fractions in seaweed extracts have also been associated with enhanced yield parameters in pea, including increased pod number, seed number and seed weight, compared with untreated plants [127]. Foliar applications have been reported to improve nutrient translocation from roots to shoots, optimize photosynthetic performance and support reproductive development [124].

Commercial formulations, such as Kelpak^®^ extracted from *Ecklonia maxima,* significantly increased cucumber plant height, leaf number and yield [128], while application of extracts from three different seaweed species increased eggplant yield by more than 30% relative to untreated controls [129]. In addition, microalgal biostimulants derived from *Chlorella vulgaris* enhance plant growth, yield, and drought tolerance by stimulating root and shoot development, modulating auxin-associated gene expressions, inducing stomatal closure, and promoting secondary metabolite biosynthesis [130]. Similarly, application of *Arthrospira (Spirulina) platensis* improves growth and photosynthesis under drought by enhancing non-enzymatic and enzymatic antioxidant capacity and increasing soluble metabolites [131]. Although these responses are often described agronomically, they are underpinned by coordinated changes in redox regulation and hormone signaling.

Depolymerization of seaweed polysaccharides, including alginates, laminarin and carrageenans, generates bioactive oligosaccharides that function as damage-associated molecular patterns (DAMPs) [132]. These marine-derived oligosaccharides trigger rapid apoplastic ROS bursts, activate SA-, jasmonic acid (JA)-, ethylene (ET)-, and ABA-dependent signaling pathways, and induce defense and antioxidant enzymes, thereby enhancing resistance to both biotic and abiotic stresses [126,133]. For example, laminarin, a β-1,3-glucan from brown algae, induces rapid ROS production and MAPK activation in treated plants [134,135]. These early signaling events resemble DAMP- or microbe-associated molecular pattern (MAMP)-triggered responses and involve activation of NADPH oxidases (RBOHs). Importantly, such ROS bursts are transient and tightly regulated, consistent with signaling functions rather than oxidative damage. The extracts derived from the red seaweed *Kappaphycus alvarezii*, rich in phenolics and κ-carrageenan, exhibit strong antioxidant and metal-chelating activity and have been reported to enhance plant growth and nutrient uptake, supporting their role in modulating ROS homeostasis and contributing to improved physiological performance under adverse environmental conditions [136,137]. This controlled oxidative activation aligns with priming mechanisms, in which plants exhibit faster and stronger responses upon subsequent stress exposure. Thus, seaweed extracts can act upstream of RBOH activation and redox-sensitive signaling cascades.

Beyond early ROS signaling, seaweed extracts reinforce antioxidant capacity. Studies in tomato and soybean report increased activities of antioxidant enzymes and enhanced proline accumulation under salinity and drought following seaweed treatment [106,138,139]. These physiological changes are associated with reduced lipid peroxidation and improved cellular redox balance. Although many mechanistic studies are conducted under controlled environments, field-based evidence confirms agronomic relevance. A two-year field trial in pepper and eggplant demonstrated consistent yield improvements following application of the *Ascophyllum nodosum* formulation SuperFifty, highlighting the importance of timing and application strategy for optimal stress resilience [140].

Extracts from the brown seaweed *Gongolaria barbata* (syn. *Cystoseira barbata*) have recently been shown to exert clear biostimulant effects in crops under non-stressful conditions. In soil-grown tomato, extracts of *G. barbata* markedly enhanced vegetative growth and fruit yield by about 65% compared with the untreated control while also improving fruit nutritional quality by raising soluble carbohydrates, phenolics, antioxidant capacity, lycopene, β-carotene, vitamin C and reduced glutathione levels [125]. In wheat, various *G. barbata* extracts significantly stimulated seedling growth, shoot length and fresh biomass by up to roughly 20–25% relative to control plants [141]. Together, these studies indicate that *G. barbata* extracts can enhance growth, yield and antioxidant metabolism in tomato and wheat, although their capacity to mitigate explicit abiotic stresses has yet to be systematically evaluated.

Among brown algae, *Ascophyllum nodosum* is one of the most extensively studied sources of plant biostimulants [106]. Extracts from this species are effective at low doses and primarily act through modulation of plant physiology via signal transduction, redox regulation, hormone interactions and metabolic adjustment [142]. Transcriptomic and physiological studies have demonstrated activation of ROS-related genes, including components regulating RBOH activity and antioxidant pathways, particularly under drought and oxidative stress conditions [143,144,145]. Increasing evidence supports the view that seaweed biostimulants function as priming agents, inducing long-lasting metabolic and transcriptional changes that enhance stress responsiveness upon later exposure [21].

Transcriptomic analyses further show that seaweed extracts modulate genes associated with antioxidant activity, osmolyte biosynthesis and hormone signaling. In *Arabidopsis thaliana*, treatment with an *Ascophyllum nodosum* extract altered the expression of stress-responsive transcription factors and ROS-scavenging enzymes [146]. Combined metabolomic and transcriptomic profiling revealed activation of ABA-responsive genes, accumulation of osmoprotectants and enhanced antioxidant capacity [143,144]. Organ-specific and time-dependent metabolic shifts were observed in both tomato and *Arabidopsis*, including changes in carbon and nitrogen metabolism [146,147]. These data suggest that seaweed biostimulants can reconfigure source–sink relations and metabolic priorities, which may contribute to yield stability under stress conditions.

Seaweed-derived biostimulants also influence hormone balance, including ABA, cytokinin, auxin, gibberellin and ethylene pathways, thereby integrating redox and hormonal control of stomatal conductance, root architecture and stress tolerance [113]. Seaweed-induced ABA accumulation under drought and modification of auxin-dependent root traits have been documented in multiple systems [126,148]. Enhanced root architecture improves nutrient and water uptake and may increase anchorage under environmental stresses, such as drought or waterlogging [124]. For instance, foliar applications of *Laminaria digitata* have shown to enhance drought tolerance in tomato by improving photosystem II efficiency, modulating stomatal conductance, water use efficiency, ABA levels, and regulating ROS detoxification pathways, resulting in improved stem water potential, reduced lipid peroxidation, and stabilization of photosynthetic pigments under water-limited conditions [149,150]. Because ABA-induced stomatal closure depends on RBOH-mediated ROS production in guard cells, seaweed extracts likely influence guard cell redox signaling rather than bypassing endogenous pathways. It is shown that *Ecklonia maxima* exerts auxin-like activity, stimulating root elongation, seminal root formation and shoot growth in maize and mung bean [151]. Moreover, seaweed-based formulations enriched in cytokinin-like compounds increase endogenous cytokinin levels in leaves and delay stress-induced senescence, as demonstrated in creeping bentgrass and spinach treated with extracts from *Ascophyllum nodosum* or *Ecklonia maxima* [152,153]. Since elevated ethylene promotes leaf senescence and ethylene inhibitors delay heat-induced senescence in cool-season grasses, it is often proposed that seaweed-derived cytokinins help to maintain a favorable cytokinin:ethylene balance during stress [154]. However, direct measurements of reduced ethylene production following seaweed application remain limited. Although cytokinins, auxins and gibberellin-like compounds have been detected in some seaweed extracts, the current consensus indicates that their primary action is modulation of endogenous hormone biosynthesis and signaling networks rather than direct hormonal supplementation.

Together, the available evidence indicates that seaweed extracts operate through controlled activation of ROS signaling, reinforcement of antioxidant systems and coordinated modulation of ABA-, auxin- and cytokinin-associated pathways. By influencing both early ROS generation and downstream hormone-regulated transcriptional responses, seaweed-derived biostimulants integrate into existing redox–hormone networks and enhance stress resilience through signaling reprogramming rather than simple nutritional enhancement (Figure 3).

### 4.3. Humic and Fulvic Substances as Redox–Hormone Regulation

Humic and fulvic substances are major components of soil organic matter and are widely recognized as natural plant growth promoters and biostimulants [19]. They are heterogeneous supramolecular associations of aromatic and aliphatic structures bearing carboxyl, phenolic and other reactive functional groups, enabling interactions with root surfaces, plasma membranes and cell walls [19]. Numerous studies show that humic fractions from soils, vermicompost and leonardite enhance root growth, nutrient uptake and stress tolerance across diverse crop species [18,19,20]. Importantly, current mechanistic models indicate that humic and fulvic substances act primarily through modulation of hormone- and ROS-related signaling pathways rather than functioning as simple fertilizers supplying nutrients.

At the molecular level, a proteomic analysis of *Arabidopsis* roots treated with humic substances revealed changes in proteins associated with energy metabolism, ROS detoxification and cell division, suggesting that their growth-promoting effects may involve auxin-like activity [155]. Integrating multiple datasets, Jespersen et al. proposed a conceptual framework in which rhizospheric humic substances regulate root and shoot growth through coordinated effects on ROS generation and scavenging, membrane transport and nutrient uptake [154]. Similarly, García et al. emphasized that ROS and hormone pathways represent the two principal signaling systems targeted by humic substances [156]. According to this view, humic-induced shifts in ROS and hormone balance within roots are translated into systemic adjustments in shoot growth, nutrient allocation and stress responses [157].

This signaling-based interpretation is supported by several mechanistic studies focused on root development. In maize and *Arabidopsis*, humic acids stimulated plasma membrane H^+^-ATPase activity and promoted lateral root formation [107,158]. These responses involved auxin signaling, whose downstream effects required NO and ROS as secondary messengers [108]. In basil hairy roots, vermicompost-derived humic acid increased root growth together with NO and ROS levels; these effects were reversed by an NO scavenger, indicating that both NO and ROS act as essential mediators of humic acid bioactivity [159]. Such findings suggest that humic substances do not simply scavenge ROS but rather induce controlled ROS and NO production that participates in developmental signaling. Agronomic evidence further supports this mechanistic interpretation. Humic acid-enriched vermicompost applied to *Brassica napus* increased root weight, seed weight, chlorophyll content and yield, consistent with improved physiological performance under field conditions [160]. Recent work extends these observations beyond root-localized responses, where foliar application of fulvic acid in rice triggered transient ROS accumulation in leaves, accompanied by increased activities of antioxidant enzymes and modulation of hormone-related gene expression. These results led to the proposal that fulvic acids drive coordinated redox and hormonal adjustments that enhance stress acclimation [161]. The transient nature of ROS accumulation is particularly important as it indicates signaling activation rather than oxidative damage.

Humic and fulvic substances also influence redox homeostasis and hormone signaling under abiotic stress conditions. Numerous studies report that humic treatments increase activities of antioxidant enzymes, reduce lipid peroxidation and improve nutrient status in plants exposed to drought, salinity or nutrient limitation [156]. Nardi et al. further concluded that humic fractions enhance antioxidant capacity and modulate redox-sensitive proteins in both leaves and roots, thereby supporting improved performance under stress [109]. More recent investigations have strengthened the mechanistic link between humic substances, ROS and hormone pathways, where chemically modified humic derivatives from vermicompost activated the auxin-responsive reporter DR5::GUS in *Arabidopsis*, suggesting that humic matter may preserve and release auxin-like molecules within hydrophobic domains [162]. This observation supports the idea that humic substances can act as carriers or modulators of auxin-like compounds, thereby influencing auxin signaling in root tissues. However, responses are not uniformly stimulatory. In rice, vermicompost-derived humic acid induced root growth accompanied by accumulation of superoxide (O_2_^•−^) and hydrogen peroxide (H_2_O_2_) in root tips while simultaneously suppressing components of auxin and ABA signaling pathways [163]. These findings indicate that humic substances can fine-tune ROS and hormone signaling networks in a context-dependent manner rather than uniformly enhancing or suppressing specific pathways.

Overall, the available evidence supports the view that humic and fulvic substances function as complex regulators of the redox–hormone network. They induce controlled ROS and NO signals in roots, modulate auxin- and ABA-related responses, enhance antioxidant capacity, and influence nutrient uptake and shoot metabolism. Rather than directly providing nutrients, humic-based biostimulants integrate into endogenous signaling circuits, adjusting ROS amplitude, hormone sensitivity and downstream transcriptional programs. These actions parallel the roles of endogenous metabolites, such as phenolics, peptides and glutathione, as described in Section 3 in Figure 2 and Figure 3, reinforcing the concept that humic and fulvic substances enhance plant performance primarily by tuning existing redox–hormone regulatory networks.

### 4.4. Higher Plant Extracts as Redox–Hormone Regulation

Extracts derived from higher plants represent a chemically diverse class of biostimulants obtained from leaves, seeds, fruits or whole aerial biomass of terrestrial species [21,115]. These extracts originate from vascular plants and typically contain phenolic compounds, flavonoids, amino acids, sugars, organic acids, vitamins and, in some cases, measurable amounts of phytohormones or hormone precursors. Common botanical sources include *Moringa oleifera*, *Medicago sativa*, *Urtica dioica* (nettle), *Glycine max* (soybean), *Vitis vinifera* (grape), *Citrus* spp., and various medicinal or aromatic plants [112,164,165]. Unlike seaweed or humic products, higher plant extracts are chemically heterogeneous and less standardized, but they consistently improve growth, yield and stress tolerance in crops [165].

Most experimental evidence indicates that their primary mode of action involves modulation of cellular redox balance. Aqueous and hydroalcoholic extraction preserves soluble secondary metabolites, particularly phenolics and flavonoids [115]. These compounds are known regulators of ROS homeostasis, capable of both direct ROS scavenging and regulation of redox-sensitive gene expression [56,57]. Because ROS act as second messengers in auxin, ABA and cytokinin signaling pathways, extract-induced changes in ROS amplitude are likely to influence downstream hormonal responses. Neem, grape byproducts and rosemary provide clear examples of higher plant extracts that modulate ROS-related responses under abiotic stress. Foliar application of neem leaf extract to drought-stressed quinoa and arsenic stressed rice seedlings enhanced shoot and root growth, further increased proline, glycinebetaine and phenolics, boosted antioxidant activities, and significantly lowered H_2_O_2_ and relative membrane permeability, indicating increased antioxidant system and membrane protection under stress [166,167]. Foliar grape seed extract from *Vitis vinifera* byproducts improved growth of salinity-stressed faba bean, reduced MDA and H_2_O_2_ accumulation, enhanced total antioxidant capacity and upregulated photosynthesis-related genes while downregulating stress-response genes, pointing to coordinated redox and metabolic reprogramming [168]. It was also shown that grape seed extract alone significantly increased the number of inflorescences per *Spathiphyllum wallisii* plant [169]. Likewise, essential oil from *Salvia rosmarinus* applied to durum wheat seedlings mitigated 150 mM NaCl stress by reducing leaf H_2_O_2_, O_2_•^−^ and MDA, strongly stimulating SOD, CAT and POD activities, and inducing transcription of Na^+^ transporters, antioxidant genes, and the gibberellin biosynthetic gene, thereby improving osmotic adjustment, photosynthetic efficiency and overall salt tolerance [170]. Together, these studies show that higher plant extracts can alleviate drought or salinity mainly by attenuating ROS accumulation, enhancing antioxidant defenses, and, in some cases, modulating growth- and stress-related gene expression within existing hormone-sensitive networks.

Additional studies support this redox-centered mechanism, where application of extracts derived from plants like *Moringa oleifera* and *Medicago sativa* increased antioxidant activities in maize, wheat, rice and bean exposed to drought or salinity, accompanied by stimulated growth, nitrogen metabolism, reduced lipid peroxidation, improved leaf water status and increased plant biomass [111,112,169,171]. Moreover, application of *Moringa oleifera* leaf extract has been associated with altered endogenous ABA levels and improved stomatal regulation in drought-stressed wheat and maize, suggesting interaction with ABA-dependent signaling pathways [111,171,172]. Similarly, plant-derived extracts applied to tomato and lettuce under salinity enhanced SOD, CAT and APX activities while lowering MDA accumulation [117]. Extracts from grape skin and seeds rich in flavonoids and proanthocyanidins improved antioxidant status and reduced oxidative damage in stressed plants [173]. These responses are consistent with controlled modulation of ROS production followed by reinforcement of antioxidant activity. Further support comes from studies under heavy metal stress, where treatment of *Capsicum annuum* grown in contaminated soils with *moringa* seed and licorice root extracts increased proline, soluble sugars and antioxidant enzyme activities [174], indicating coordinated metabolic and redox adjustments. In lettuce under salinity, *moringa* leaf extract improved growth and yield while reducing electrolyte leakage and sodium accumulation [172], again associated with enhanced antioxidant status.

Direct transcriptomic or metabolomic evidence demonstrating modulation of hormone biosynthesis or signaling pathways by higher plant extracts remains limited. However, given the central role of ROS in mediating ABA-induced stomatal regulation and auxin-dependent root development, extract-driven redox adjustments are expected to influence hormone sensitivity and signal propagation. Phenolic constituents may further affect auxin transport and distribution, providing a mechanistic basis for the observed changes in root architecture and growth responses reported in several crops. Collectively, the available data support the view that higher plant extracts function primarily as modulators of the redox network, with secondary effects on hormone-regulated processes emerging through established ROS–hormone crosstalk. Rather than acting as direct hormone substitutes, these extracts appear to adjust redox buffering capacity and signaling thresholds within endogenous regulatory circuits. In this respect, their mode of action parallels that described for humic substances and algae-derived biostimulants, reinforcing the concept shown in Figure 1 and Figure 3 that external plant-based products enhance stress resilience by tuning existing redox–hormone signaling networks rather than introducing novel pathways.

**Table 2:** Species of terrestrial plants and algae used in biostimulant production and their effects on crops.

Source Species	Biostimulant Class	Dominant Bioactive Fractions	Effects on Crops	References
*Arthrospira platensis* (syn. *Spirulina*)	Microalgal hydrolysate	Amino acids, peptides, pigments	Improves growth & photosynthesis, enhances non-enzymatic & enzymatic antioxidant capacity, & increases soluble metabolites	[130,131]
*Ascophyllum nodosum* (Brown alga)	Seaweed extract	Alginates, laminarin, betaines, phenolics	Improves photosynthesis, antioxidant activity, root/shoot growth, & yield	[138,145]
*Azadirachta indica* (Neem)	Higher plant extract	Limonoids, flavonoids, phenolics	Enhances shoot & root growth, increases proline, glycine betaine, phenolics & antioxidant activities, & protects membrane integrity	[166,167]
*Chlorella vulgaris* (Green microalga)	Microalgal extract	Proteins, amino acids, vitamins, antioxidants	Enhances plant growth & yield, stimulates root & shoot development, regulates stomatal movement & promotes secondary metabolite biosynthesis	[130]
*Ecklonia maxima* (Brown alga)	Seaweed extract	Phlorotannins, auxin-like compounds	Stimulates early growth, root elongation & shoot growth, & delays senescence	[151,152]
*Gongolaria barbata* (Brown alga)	Seaweed extract	Phlorotannins, polysaccharides, phenolics	Enhances vegetative growth & fruit yield, improves fruit nutritional quality & antioxidant capacity, & stimulates shoot length & fresh biomass	[125,141]
*Kappaphycus alvarezii* (Red alga)	Seaweed extract	Carrageenans, sulfated polysaccharides	Enhances nutrient uptake, antioxidant & metal-chelating activity, & modulates ROS homeostasis	[136,137]
*Laminaria digitata* (Brown alga)	Seaweed extract	β-glucans (laminarin), polysaccharides	Enhances photosystem II, improves stomatal regulation & water potential, promotes ROS detoxification, & reduces lipid peroxidation	[126,149,150]
*Medicago sativa* (Alfalafa)	Higher plant extract	Amino acids, peptides, phenolics	Stimulates growth, nitrogen metabolism & plant biomass; reduces lipid peroxidation; improves leaf water status	[112,121,169]
*Moringa oleifera*	Higher plant extract	Phenolics, flavonoids, vitamins, amino acids	Enhances antioxidant activity & soluble sugars, reduces lipid peroxidation & electrolyte leakage, & improves leaf water status, growth & yield.	[171,172]
*Salvia rosmarinus* (Rosemary)	Higher plant extract	Phenolics, terpenoids, flavonoids	Stimulates antioxidant activity, induces Na^+^ transporters, improves osmotic adjustment & enhances photosynthetic efficiency	[170]
*Vitis vinifera* (Grape byproduct)	Higher plant extract	Flavonoids, proanthocyanidins	Improves growth, increases inflorescence development, reduces oxidative stress, and enhances antioxidant capacity & photosynthesis	[168,173,175]

Only the species mentioned in the text are shown.

## 5. Conclusions

ROS and phytohormones constitute a central regulatory framework governing plant adaptation to abiotic stress. As outlined in Section 3, endogenous metabolites and signaling molecules, including phenolics, terpenoids, alkaloids, small peptides, glutathione and polyamines, function within interconnected regulatory nodes rather than as isolated antioxidants. They converge on defined components, such as NADPH oxidases (RBOHs), antioxidant enzyme systems, redox-sensitive transcription factors and hormone signaling cascades. Through modulation of ROS production, redox buffering capacity and hormone sensitivity, these endogenous factors shape the amplitude, duration and spatial distribution of stress signals. Section 4 extends this framework to terrestrial plant- and algae-derived biostimulants. Across PH, seaweed extracts, humic and fulvic substances, and higher plant extracts, a consistent mechanistic pattern emerges. These external inputs integrate into pre-existing ROS–hormone networks by influencing RBOH activity, antioxidant capacity, membrane-associated transport processes and hormone-responsive gene expression. In several cases, transient ROS accumulation precedes enhancement of the antioxidant system, supporting a signaling-based mode of action rather than direct oxidative mitigation. Modulation of the ABA-, auxin-, ethylene- and cytokinin-associated pathways appears to occur through established redox-sensitive regulatory nodes. Figure 3 summarizes this evidence into a unified model in which abiotic stresses, endogenous metabolites and exogenous biostimulants converge on shared ROS–hormone hubs. The primary outcome is quantitative tuning of signal intensity, buffering capacity and hormone responsiveness within the existing regulatory architecture. Within this framework, biostimulant efficacy is expected to depend on the physiological state of the plant, the nature and severity of the stress, and the redox–hormone equilibrium at the time of application.

## 6. Knowledge Gaps and Future Directions

Despite the rapid expansion of the biostimulant market, meta-analyses and recent reviews consistently emphasize that product efficacy remains highly variable across crops, environments and formulations [176]. A global meta-analysis of more than 1000 open-field comparisons reported an average yield increase of 17.9% for non-microbial biostimulants but also detected substantial heterogeneity (*I*^2^ ≥ 75%) and stronger responses in low-fertility or stress-prone soils, indicating that performance is strongly context-dependent [177]. For humic substances, field studies show that responses depend on molecular composition, extraction method and dose, and that commercial application rates are often an order of magnitude lower than concentrations that are effective in greenhouse assays, which can explain inconsistent agronomic outcomes [178]. Seaweed-based products exhibit similar variability as chemodiversity among species, seasonal changes in bioactive constituents and differences in extraction processes all generate extracts with divergent biological activities [179]. These observations highlight the need for tighter standardization of raw materials, extraction protocols and labeling, as well as harmonized testing frameworks.

The mechanistic basis of the widely cited “auxin-like” activity of humic substances remains debated, representing an important knowledge gap. Some studies report that humic fractions contain low levels of IAA, stimulate lateral root formation and activate auxin-responsive genes, with these effects being absent in auxin-insensitive mutants, indicating dependence on functional auxin signaling [156,180]. In contrast, other studies show that certain humic fractions can modify root architecture without strong activation of auxin reporters or rescue of auxin-related mutants, suggesting auxin-independent mechanisms [156,180]. Together, these findings indicate that humic “auxin-like” activity cannot yet be attributed unambiguously to exogenous auxin analogues and highlight the need for better-characterized fractions and targeted use of auxin signaling mutants. Similarly, several seaweed-based biostimulant studies highlight that multiple bioactive classes act in combination, and transcriptomic and mutant analyses point to priming of ROS, autophagy and hormone-responsive pathways; however, these signaling pathways have not yet been assembled into a comprehensive model or validated across different seaweed-based biostimulant products. More generally, several authors identify insufficient mechanistic understanding and a lack of targeted mutant and functional-genomics studies as major bottlenecks for moving from empirical biostimulant use towards rational “next-generation” formulations that deliberately address defined signaling hubs.

Future research should combine transcriptomic, metabolomic and redox profiling approaches to define stress-specific ROS signatures, glutathione dynamics, RBOH activity patterns and hormone signaling thresholds. Such integrative analyses will be necessary to distinguish between general reinforcement of redox buffering and targeted modulation of defined hormone circuits. Embedding these approaches within multi-year multi-site field trials will be essential to link molecular responses to robust agronomic outcomes. A system-level understanding of these shared regulatory hubs, as outlined in our conceptual framework (Figure 1 and Figure 3), will enable more precise mechanistic interpretation and support rational optimization of biostimulant use under field conditions.

## Figures and Tables

**Table 1:** Representative biostimulant categories, their key bioactive compounds, primary physiological effects and ROS–hormone modulation under abiotic stress.

Biostimulant Category	Key Bioactive Components	Primary Physiological Effect	ROS–Hormone Modulation	References
Protein hydrolysates	Free amino acids, oligopeptides, small peptides	Improved biomass, root architecture, maintenance of photosynthesis	Enhanced antioxidant, auxin-, GA-, cytokinin-, ethylene-signaling	[19,20,21,110]
Seaweed extracts	Alginates, laminarin, carrageenans, fucoidans, betaines, polyphenols	Root growth, enhanced yield, osmotic adjustment, stomatal regulation, improved photosynthesis antioxidant enhancement	RBOH activity, ABA-, auxin-, JA-, cytokinin-, ethylene- signaling, enhanced antioxidant	[19,21,113]
Humic/fulvic substances	Humic acids, fulvic acids, phenolic fractions	Root and shoot growth, improved ion homeostasis, membrane stability, enhanced nutrient uptake	Plasma membrane, H^+^-ATPase activity, auxin-like effects, ABA interaction	[19,20,114]
Higher plant-derived extracts	Phenolics, terpenoids, oligosaccharides, flavonoids	Growth enhancement, reduced lipid peroxidation, reduced electrolyte leakage	Cellular redox balance, cytokinin-, ABA-signaling	[21,115]

## Data Availability

No new data were created or analyzed in this study.
